# Molecular Determinants of the Promiscuity of MexB and MexY Multidrug Transporters of *Pseudomonas aeruginosa*

**DOI:** 10.3389/fmicb.2018.01144

**Published:** 2018-06-01

**Authors:** Venkata K. Ramaswamy, Attilio V. Vargiu, Giuliano Malloci, Jürg Dreier, Paolo Ruggerone

**Affiliations:** ^1^Department of Physics, University of Cagliari, Monserrato, Italy; ^2^Basilea Pharmaceutica International Ltd., Basel, Switzerland

**Keywords:** RND efflux pumps, multidrug transporter, *Pseudomonas aeruginosa*, antibiotic resistance, molecular dynamics, molecular modeling

## Abstract

Secondary multidrug transporters of the resistance-nodulation-cell division (RND) superfamily contribute crucially to antibiotic resistance in Gram-negative bacteria. Compared to the most studied transporter AcrB of *Escherichia coli*, little is known about the molecular determinants of distinct polyspecificities of the most important RND transporters MexB and MexY of *Pseudomonas aeruginosa*. In an effort to add knowledge on this topic, we performed an exhaustive atomic-level comparison of the main putative recognition sites (access and deep binding pockets) in these two Mex transporters. We identified an underlying link between some structural, chemical and dynamical features of the binding pockets and the physicochemical nature of the corresponding substrates recognized by either one or both pumps. In particular, mosaic-like lipophilic and electrostatic surfaces of the binding pockets provide for both proteins several multifunctional sites for diffuse binding of diverse substrates. Specific lipophilicity signatures of the weakly conserved deep pocket suggest a key role of this site as a selectivity filter as in Acr transporters. Finally, the different dynamics of the bottom-loop in MexB and MexY support its possible role in binding of large substrates. Our work represents the first comparative study of the major RND transporters in *P. aeruginosa* and also the first structure-based study of MexY, for which no experimental structure is available yet.

## Introduction

*Pseudomonas aeruginosa* is an opportunistic human pathogen and a leading cause of nosocomial infections worldwide due to the emergence and spread of multi, extensive, and pan-drug resistant isolates susceptible to very few antimicrobial agents (Fischbach and Walsh, 2009; Poole, 2011). The intrinsic resistance of *P. aeruginosa* to multiple antibiotics results from the synergy between its low permeable outer membrane and the action of (chromosomally encoded) multidrug efflux systems like the ones constituted by the resistance-nodulation-cell division (RND) superfamily of secondary transporters (Hancock, 1998; Li et al., 2015), which contribute to both intrinsic and acquired resistance (Poole, 2001; Poole and Srikumar, 2001; Dreier and Ruggerone, 2015). Several RND type efflux systems have been identified in *P. aeruginosa* PAO1 (Webber and Piddock, 2003; Poole, 2005; Lister et al., 2009; Zechini and Versace, 2009; Fernández and Hancock, 2012; Blair et al., 2014; Delmar et al., 2014; Sun et al., 2014), with the most significant for multidrug resistance being MexAB-OprM (Poole et al., 1993; Gotoh et al., 1995) and MexXY-OprM (Aires et al., 1999; Mine et al., 1999; Westbrock-Wadman et al., 1999). These two machineries contribute additively to the resistance to common substrate antibiotics (Lee et al., 2000; Llanes et al., 2004); moreover, their different specificities (viz. MexB for β-lactams and MexY for aminoglycosides) drastically reduce the susceptibility of infectious strains to numerous classes of antibiotics (Llanes et al., 2004).

**FIGURE 1:**
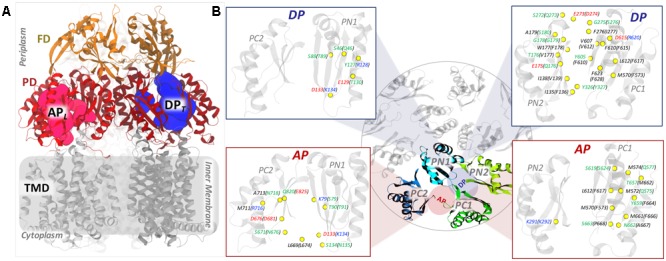
General structure of an RND transporter and comparison of the putative binding pockets (AP and DP) between MexY and MexB. **(A)** The general structure of an RND transporter highlighting the three main domains (TMD, PD, and FD) with different colors. **(B)** The figure in the middle shows the top view of the four main domains (colored differently) enclosing the AP and DP. The locations of the pockets are schematically shown as red and blue colored circles for AP and DP, respectively. The insets highlight the mismatched residues of MexY and MexB as yellow-colored beads with the residue labels colored by residue type (non-polar residues in black, polar residues in green, basic residues in blue, and acidic residues in red). The residue labeling follows the notation “MexY (MexB).”

The MexAB-OprM tripartite system was the first RND-type multidrug efflux system to be discovered in *P. aeruginosa* at approximately the same time as the AcrAB-TolC system of *E. coli* (Poole et al., 1993). MexB resembles AcrB with a jellyfish-like structural topology formed by an asymmetric trimer with each protomer comprising three domains (Ruggerone et al., 2013) (**Figure 1A**): (i) a trans-membrane domain (TMD) of 12 α-helices embedded in the inner membrane (IM), where the chemical-to-mechanical energy conversion takes place; (ii) a pore (porter) domain (PD) located in the periplasm, where substrate recruitment and transport occur; and (iii) a periplasmic funnel domain (FD), which connects the RND transporter to the outer membrane protein (OMP) via the assembly of membrane fusion proteins (MFPs) (Symmons et al., 2015) in the constituted pump. Substrate transport is characterized by the typical “functional rotation mechanism” (Supplementary Figure 1) in which concerted (but not necessarily synchronous) cycling of the protomers occurs through all of the so far identified asymmetric states: *Loose (L)* (a.k.a. *Access*) in which a substrate binds to a peripheral site termed access pocket (AP*_L_*); *Tight (T)* (a.k.a. *Binding*) in which the substrate binds to a deeper pocket (DP*_T_*); and *Open (O)* (a.k.a. *Extrusion*) in which the substrate is released into the central funnel leading toward the OMP (Murakami et al., 2006; Seeger et al., 2006; Pos, 2009). The two pockets, AP*_L_* and DP*_T_* (**Figure 1B** and Supplementary Figure 1), were previously identified in AcrB [and the latter also in MexB (Nakashima et al., 2013)] as the binding sites responsible for the recognition and selectivity of different types of substrate molecules based on their molecular weight or chemical type (Nakashima et al., 2011; Kobayashi et al., 2014; Iyer et al., 2015; Schuster et al., 2016). The pockets are separated by a G-rich (a.k.a. switch) loop whose flexibility has been shown to be important for the transport of high-molecular mass compounds (Nakashima et al., 2011; Eicher et al., 2012).

The MexY system, identified later, shares an overall sequence identity (similarity) of nearly 47% (66%) with MexB and nearly 48% (67%) with AcrB and AcrD (Supplementary Table 1). Thus, MexY is expected to resemble them in global features like structural fold, location of main ligand binding pockets and functional rotation mechanism (Srikumar et al., 1997; Murata et al., 2002; Eda et al., 2003a). However, MexB and MexY display relevant differences in their substrate specificities (**Table 1** and Supplementary Figures 2, 3). For instance, the substrate specificity of MexB is very similar to that of AcrB (e.g., both proteins transport macrolides such as erythromycin, most beta-lactams, chloramphenicol, etc.) and slightly yet significantly different from that of MexY (aminoglycosides such as gentamicin, tobramycin, amikacin, and isepamycin are transported only by MexY but not by MexB and AcrB) (Krahn et al., 2012; Dreier and Ruggerone, 2015).

**Table 1:** Antibiotic substrate specificities of the paralog RND transporters MexB and MexY from *P. aeruginosa* (Li et al., 1995; Köhler et al., 1996; Zhao et al., 1998; Ziha-Zarifi et al., 1999; Masuda et al., 2000a; Chuanchuen et al., 2001; Okamoto et al., 2001; Hocquet et al., 2003; Llanes et al., 2004; Mesaros et al., 2007; Nakashima et al., 2013).

Transporter(s)	MexB	MexY	MexB and MexY
Substrates	Most beta-lactams (except imipenem), novobiocin, trimethoprim and triclosan	Aminoglycosides (gentamicin, tobramycin, amikacin), Penicillins (except carbenicillin and sulbenicillin), Cephems (except cefsulodin and ceftazidime)	Macrolides (erythromycin, spiramycin), Fluoroquinolones, chloramphenicol, Tetracyclines
Substrate type	Hydrophobic	Hydrophilic	Amphiphilic

*Classes of compounds are indicated, with examples of specific compounds within parentheses (2D chemical structures of these compounds are shown in Supplementary Figure 2). General physicochemical property of antibiotic substrates of MexB and MexY are also specified.*

Previous studies on these Mex pumps focused on identifying domains responsible for substrate recognition by means of chimeric domain swapping (Tikhonova et al., 2002; Eda et al., 2003b). A few investigations attempted to identify the substrates of Mex pumps (Masuda et al., 2000b; Collu et al., 2012), the residues involved in substrate selectivity (Middlemiss and Poole, 2004; Wehmeier et al., 2009) and also to explain the structural basis for the differential binding of inhibitors to MexB and MexY (Nakashima et al., 2013). However, the molecular basis for the diversity in the substrate profile of these Mex pumps remains largely unknown. One of the key steps to bridge this gap would be to map the differences in substrate specificities between these proteins to distinct structural, chemical and dynamic features of their main putative substrate-binding pockets. Unfortunately, the absence of an experimental structure of MexY and the availability of only one structure of MexB bound to compounds [the ABI-PP inhibitor D13-9001 within DP*_T_* (Nakashima et al., 2013)] have made it hard to reach this goal. However, given the overall good sequence identity and similarity of MexY with MexB of *P. aeruginosa* and with AcrB of *E. coli* for which high resolution X-ray structures are available, reliable computational modeling of MexY and related structure-based studies are possible.

In addition, as these biological systems are not static *in vivo*, understanding their dynamics in terms of statistically relevant conformations, interactions with solvent, and physicochemical nature of the putative binding pockets is essential for a more robust comparison. In this regard, computational modeling, in particular all-atom molecular dynamics (MD) simulations, have already proven to be valuable in addressing the molecular mechanisms of RND transporters (Schulz et al., 2010; Vargiu et al., 2011, 2014, 2018; Collu et al., 2012; Fischer and Kandt, 2013; Ruggerone et al., 2013; Zuo et al., 2015, 2016; Ramaswamy et al., 2017a,b; Matsunaga et al., 2018). By employing homology modeling and extensive multi-copy μs-long MD simulations, we recently identified the underlying link between the microscopic environment of the dynamic binding pockets and drug properties that governs and regulates substrate recognition and transport in AcrB and AcrD transporters of *E. coli* (Ramaswamy et al., 2017b). Very little is known of the physicochemical and dynamic features of the corresponding binding pockets in MexB and hardly anything for MexY.

In this work, we characterized and compared the main putative binding pockets (AP*_L_* and DP*_T_*) of MexB and MexY in terms of molecular descriptors like accessible volume, lipophilic index, electrostatic potential, hydration profile and distribution of multi-functional sites (MFSs). These descriptors depend on the sequence, structure and dynamics of the pockets, and clearly affect the molecular recognition of substrates. We identified features that potentially explain the highly multifunctional nature of these pockets in MexB and MexY. In particular, the ability of MexY to accommodate a very diverse set of substrates ranging from hydrophobic macrolides to hydrophilic aminoglycosides, can be explained by the intermediate lipophilic profile (scaling between that of AcrB and AcrD) in synergy with the mosaic-like electrostatic environment of its main putative binding pockets. Furthermore, correlating our previous findings on the structure-function relation of Acr transporters (Ramaswamy et al., 2017b) with those of Mex transporters could be informative to new drug design attempts addressing efflux pumps-based antibiotic resistance (Ruggerone et al., 2013).

## Results and Discussion

### Sequence Comparison

Since bacteria respond to adverse environmental stress by altering their genetic makeup, we first analyzed the sequences of MexB and MexY from all available bacterial strains of *P. aeruginosa*. Both these protein sequences were found to be well conserved across the strains deposited in UniProtKB^1^ (accessed November 2017). MexB of *P. aeruginosa* and AcrB of *E. coli* showed a comparable sequence identity (∼47 and ∼48%, respectively) and similarity (∼66 and ∼67%, respectively) with MexY having least gaps (none in the binding pockets) over maximum sequence coverage (Supplementary Table 1). Further, on comparing MexY to MexB (**Figure 1B** and Supplementary Figure 3), we noticed that both AP and DP were less conserved than the overall proteins, sharing only around 35 and 34% identities, respectively. However, in terms of chemical composition of the pockets, the DP of both MexB and MexY showed an equal proportion of hydrophobic residues (∼50%), but different proportions of polar and charged residues (32 and 11% in MexB vs. 23 and 16% in MexY, respectively). The AP exhibited a slightly higher proportion of hydrophobic residues and a lower proportion of polar and charged residues in MexB than in MexY (56% vs. 50%, 21% vs. 27%, and 13% vs. 15%, respectively). Most of the residues identified as essential to establish interactions with the substrates and/or the inhibitors in AcrB (Elkins and Nikaido, 2002; Nakashima et al., 2011; Vargiu et al., 2011; Eicher et al., 2012; Yao et al., 2013; Kobayashi et al., 2014; Blair et al., 2015; Opperman and Nguyen, 2015) were well conserved in MexB. The characteristic hydrophobic trap (HP-trap) sitting within the DP and rich in phenylalanine residues was completely conserved in MexB but not in MexY. The HP-trap does interact only smoothly with the transported drugs (Kinana et al., 2016) but it is likely a preferred target site for inhibitor design in AcrB (Bohnert et al., 2008; Vargiu et al., 2011, 2014; Ruggerone et al., 2013; Opperman and Nguyen, 2015; Ramaswamy et al., 2016). The corresponding HP-trap region in MexY though mostly hydrophobic contains the hydroxyl group of Y605 (corresponding to F610 in MexB and AcrB) and the nitrogen atom of the indole ring of W177 (corresponding to F178 in MexB and AcrB) (Nakashima et al., 2013).

### MD Simulations of MexB and MexY

The all-atom MD simulations of apo-form of MexB were started from the crystallographic structure with PDB code 3W9I (pre-MD of MexB). As no experimental structure of MexY is available to date, we generated a reliable multi-template homology model of this transporter using the crystal structures of MexB (PDB code 3W9I) and AcrB (PDB code 4DX5). Details of the rigorous validation of this model (pre-MD of MexY) are reported in Supplementary Material (Supplementary Table 2). The stability of the MexY model and its suitability for subsequent quantitative analyses were further validated by performing 4 independent μs-long MD simulations.

Considering the root mean square deviation of the whole protein backbone and of each protomer with reference to the initial structure (Supplementary Figure 4), we established the equilibration time of ∼0.2 μs to be the most suitable for both MexB and MexY simulations. On the remaining ∼4 μs-long (4 × 1 μs) cumulative MD trajectory of each protein, we performed a cluster analysis to extract statistically relevant conformations sampled by the proteins (Supplementary Figures 5, 6). The most populated clusters were used to characterize the distribution of accessible binding volumes, molecular lipophilicity, electrostatic potential and MFSs. The trajectories themselves were further analyzed for hydration patterns within the AP*_L_* and DP*_T_* of both proteins. In the following, we discuss separately the results of these characterizations.

### Access Pocket of the *Loose* Protomer

#### Pocket Volume and Essential Dynamics

The accessible volume at the recognition pocket is the first of the many factors governing optimal ligand binding in addition to shape and electrostatic complementarity (Ruiz-Carmona et al., 2014). Promiscuous RND transporters were earlier identified to have a large binding site with a reasonable degree of plasticity to facilitate binding of molecules of a wide range of sizes (Edward et al., 2003; Nikaido and Takatsuka, 2009; Marsh, 2015).

**FIGURE 2:**
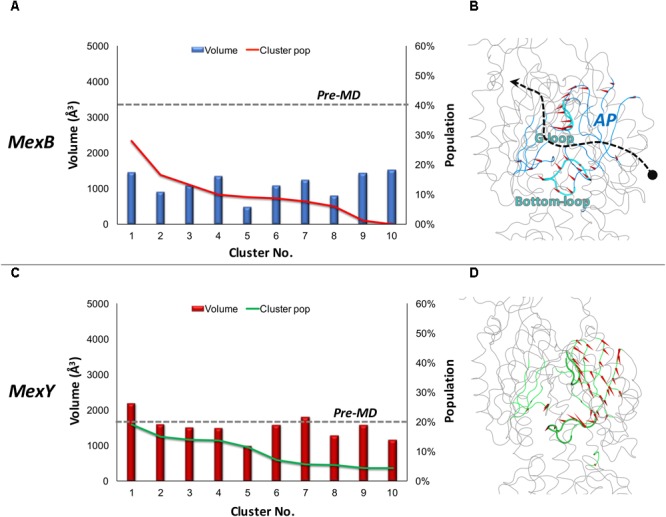
Volume dynamics of AP*_L_* of MexB and MexY. (Left panel) Distribution of the volume of AP*_L_* of MexB **(A)** and MexY **(C)**, calculated for the 10 top cluster representatives extracted from equilibrium MD trajectories. Histograms refer to the volumes, lines to the relative population of the corresponding clusters. The volumes calculated for the pre-MD structures of MexB and MexY are shown as dashed lines. (Right panel with side view of the protomer) Porcupine plots representing collective motions along the PC eigenvector for AP*_L_* in MexB **(B)** and MexY **(D)** simulations shown as arrows (>2Å) attached to Cα atoms indicating the magnitude of the corresponding eigenvalues. Features of AP*_L_* are colored blue and green in MexB and MexY, respectively. The G-loop and bottom-loop are shown as thicker tubes. The substrate path from the periplasmic entrance (dot) to the exit gate (arrow head) is shown with a dashed arrow passing between the two main loops likely governing substrate access and transport in RND pumps.

The AP*_L_* of MexB featured an average value of ∼1120 Å^3^ with the most populated cluster (about 28% of the simulation time) showing a pocket volume of around 1440 Å^3^. In the case of MexY, the AP*_L_* showed a larger average volume of ∼1590 Å^3^ with the most populated cluster (covering ∼19% of the simulation time) showing around 2180 Å^3^ (**Figure 2** and **Table 2**). Interestingly, while in MexB the volume of AP*_L_* diminished significantly from that in the pre-MD structure (3350 Å^3^), the values calculated in MexY were overall in line with the initial volume of ∼1600 Å^3^ (**Table 2**). The opening of this site in the crystal structures 2V50 (Sennhauser et al., 2009) and 3W9I (Nakashima et al., 2013) of MexB with respect to the MD-based structures could, for instance, have been induced by the presence of other (perhaps unresolved) molecules, as suggested earlier for the DP*_T_* of AcrB (Seeger et al., 2006; Sennhauser et al., 2007; Fischer and Kandt, 2013).

**Table 2:** Volumes of AP*_L_* and DP*_T_* of MexB and MexY.

System	Volume (Å^3^)	
	Pre-MD	MD clusters
**AP*_L_***		
MexB	3350	1120 ± 290
MexY	1600	1590 ± 350
**DP*_T_***		
MexB	5120	2310 ± 200
MexY	3840	2400 ± 210

*For MexB, the pre-MD structure corresponds to the crystal structure identified by PDB code 3W9I while for MexY it is the final optimized model used as starting configuration for MD simulations.*

It is also worth pointing out that both Mex transporters display smaller AP*_L_* average volumes compared to AcrB and AcrD (about 2510 ± 440 Å^3^ and 3010 ± 380 Å^3^, respectively). In particular, during MD we observed a compression of the AP*_L_* volume of nearly 66% in MexB vs. 30% in AcrB. It is to be noted that a constricted state of MexB (PDB code 2V50) with respect to AcrB was previously reported by Sennhauser et al. (2009) from their crystallographic studies. Nonetheless, the volumes of AP*_L_* in MexB and MexY (**Table 2**) are much larger than those of the largest substrates [e.g., erythromycin having a volume of 727 ± 2 Å^3^ (Malloci et al., 2015)] transported by these pumps. This indicates the possibility of a substrate to bind in different orientations and/or at different sub-pockets, a hypothesis compatible with the multisite-drug-oscillation (Yamaguchi et al., 2015) and diffuse binding (Marsh, 2015) in these proteins.

**FIGURE 3:**
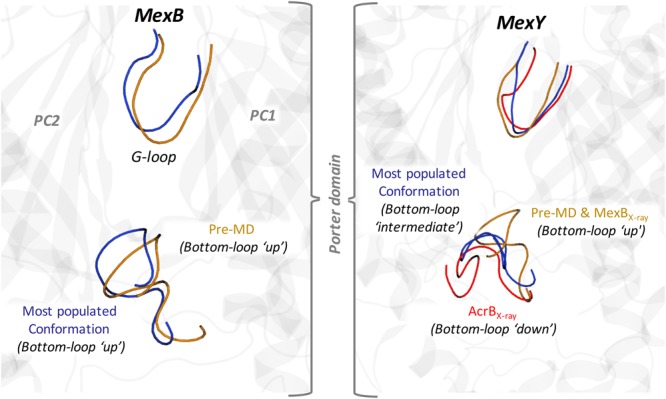
Main conformational states of the bottom-loop in MexB (Left panel) and MexY (Right panel). The conformation of the most populated MD clusters and the pre-MD structures are shown in blue and orange cartoons, respectively. The conformation of AcrB (PDB code 4DX5) is also shown for reference in red. The conformations of the G-loop are also indicated with the same color code.

In addition to pocket volume calculations, we performed principal component analysis (PCA) of equilibrium MD trajectories in order to identify the essential dynamics of regions lining the putative main binding pockets. Porcupine plots of the top three principal components (**Figures 2B,D**) show the entire AP*_L_* of MexB and MexY exhibiting almost a coherent motion with slightly larger magnitude (depicted by length of the arrows) in the case of MexB. The dynamicity of the bottom-loop lining the base of AP*_L_* and earlier identified as a peculiar feature of AcrB but not AcrD (Ramaswamy et al., 2017b) appeared to be different even in Mex transporters (**Figure 3**). The most populated cluster in MexB and in fact all cluster representatives were characterized by an “up” conformation, comparable to the pre-MD or the crystal structure of MexB (PDB code 3W9I). In the case of MexY, the corresponding bottom-loop showed an intermediate state between the “up” state of its pre-MD and its MexB template structure and the “down” state seen in the AcrB template structure (PDB code 4DX5). The magnitude of Cα displacements of the bottom-loop was different in Mex (<6.4 Å in MexB and <8.9 Å in MexY) and Acr (<12.5 Å in AcrB and <6.7 Å in AcrD) transporters (Ramaswamy et al., 2017b). On comparing the amino acid sequence among these different transporters, we found it interesting that this loop is poorly conserved across the Mex (MexB: LELGNA, MexY: PDLGST) and Acr (AcrB: VELGTA, AcrD: SGLGSS) transporters, with only residues LG being fully preserved across the four proteins. This could partly explain the differential dynamicity of the bottom-loop observed in our simulations. Further studies are needed to investigate the role of this loop in synergy with the G-loop in regulating substrate access and transport in these RND transporters.

#### Lipophilic Index (LI) and Molecular Lipophilicity Potential (MLP)

In addition to steric features, an adequate lipophilic profile is essential for suitable binding of hydrophobic or amphipathic molecules such as macrolides (e.g., erythromycin and spiramycin) transported by both Mex proteins. In order to characterize the lipophilicity of the pocket, estimate its dependence on the conformation of the protein, and compare closely related RND transporters, we calculated the LI of AP*_L_* for the pre-MD structures and for representative structures of all clusters. There was no remarkable difference in LI of AP*_L_* between pre-MD and MD values of MexB and MexY in our case [**Table 3** and Supplementary Figure 7 (Upper panel)], as observed also for the LIs of Acr proteins (Ramaswamy et al., 2017b) [AcrB: 7.2 vs. 7.0 (±1.0) and AcrD: 1.2 vs. 1.6 (±0.6) for pre-MD and MD, respectively].

**Table 3:** Lipophilic indexes of AP*_L_* and DP*_T_* of MexB and MexY.

System	Lipophilic Index	
	Pre-MD	MD clusters
**AP*_L_***		
MexB	2.7	2.7 ± 0.9
MexY	4.2	4.5 ± 1.2
**DP*_T_***		
MexB	20.1	4.1 ± 2.3
MexY	15.9	8.9 ± 2.2

*See **Table 2** for further details.*

**FIGURE 4:**
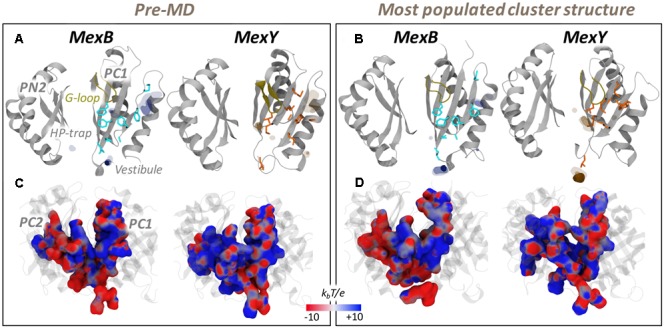
MLP and electrostatic potential of AP*_L_* of MexB and MexY. (Upper panel) MLP isosurfaces observed within 4 Å of AP*_L_* of MexB (blue) and MexY (orange) in pre-MD **(A)** and the representatives of the most populated cluster **(B)** as seen from the center of the protomer. The hydrophobic/aromatic residues are shown as sticks in the structures. Isosurfaces at 0.75 (solid), 0.5 (dark transparent), and 0.25 (light transparent) are shown in blue (MexB) or orange (MexY). The HP-trap and Vestibule sites are also labeled in the pre-MD structure of MexB. The G-loop is shown in yellow cartoon. (Lower panel) The electrostatic potential plotted on the molecular surface representation of AP*_L_* in the pre-MD **(C)** and the most populated cluster representative **(D)** of the Mex proteins as seen from the periplasmic front of the protomer. The color code is red to blue from negative (-10 k_b_T/e) to positive (+10 k_b_T/e) potential, where k_b_ is the Boltzmann constant, T is the absolute temperature and e is the electron charge.

Considering the four RND transporters, we found that MexB and MexY featured intermediate LIs between those of AcrB (highest) and AcrD (lowest). Specifically, for the Mex transporters, the LIs were slightly higher for MexY than for MexB, despite the higher percentage of hydrophobic residues at this site in the latter protein. This is due to the reduced exposed lipophilic surface (Oberhauser et al., 2014) associated with the aforementioned closure of the pocket in MexB during MD simulations. To verify this aspect we considered open structures of MexB [both crystal structures with PDB codes 2V50 (Sennhauser et al., 2009) and 3W9J (Nakashima et al., 2013), and homology models built using AcrB with PDB code 4DX5 (Eicher et al., 2012) as template] and top five models of MexY built using 2V50 as template. The average LI values computed were 6.4 ± 2.5 and 5.5 ± 1.0 for MexB and MexY, respectively, in line with the features of the residues lining the pocket. Overall, the intermediate values of the Mex transporters reveal that the specific chemical environment of their APs is neither entirely hydrophobic nor entirely polar in both proteins as noticeable from their molecular lipophilic surfaces (**Figures 4A,B**), thereby giving rise to weak binding with dispersed interactions, possibly facilitating substrate transport (Marsh, 2015; Yamaguchi et al., 2015).

#### Electrostatic Potential

The recognition of charged substrates (viz. polycationic aminoglycosides by MexY and zwitterionic or anionic β-lactams by MexB) is mediated by electrostatic complementarity, which is essential for initial substrate recruitment and augmentation of their association rate (Selzer et al., 2000; Levy et al., 2007). The AP*_L_* of MexY has a slightly greater number of polar and charged residues compared to that of MexB. This difference was mirrored in the different electrostatic potentials of the two transporters, as can be seen from their projection onto the solvent accessible surface areas in **Figures 4C,D**. In particular, two main regions are clearly visible in MexB: a negative patch near the base of AP*_L_* and on the PC2 domain, and positive patches near the zone exposed to the periplasmic cleft entrance (mostly on PC1). This separation was less intense in MexY, which compared to MexB also featured an overall greater distribution of positive patches within the AP*_L_*. The marked influence of electrostatics on substrate recognition and transport in MexY was already highlighted in an experimental mutation study reported by Poole and co-workers (Lau et al., 2014). In particular, three residues (D133, Y613, and K79) principally lining the AP compromised (D133, Y613) or enhanced (K79) aminoglycoside resistance upon substitution. These effects are in agreement with our findings, as the removal of the positive charge on K79 along the transport path likely enables a more efficient transport of molecules such as polycationic aminoglycosides, while substituting D133 with S or A, thus removing a negative patch in that pocket, probably has a negative effect on the recognition/binding of positively charged molecules.

It is interesting to note that the electrostatic nature of MexB and MexY seen here are comparable to that of AcrB (more negative) and AcrD (more positive), respectively. However, based on the homology to Acr transporters of *E. coli* in which residues essential for specificity to anionic β-lactams in AcrD were recently identified (Kobayashi et al., 2014), we found the corresponding residues (Q in MexB/MexY at position of R568 in AcrD; M in MexB/MexY at position of R625 in AcrD; E in MexB and D in MexY at position of G672 in AcrD) to differ in MexB and MexY. This lack of sequence identity may indicate a different selection filter for charged substrates in these Mex transporters.

#### Hydration Analysis

**FIGURE 5:**
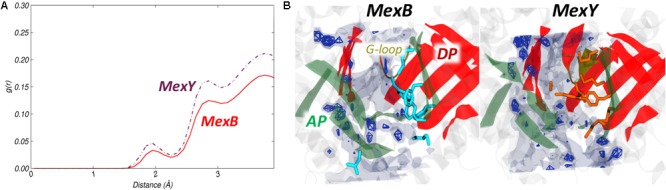
Hydration of AP*_L_* of MexB and MexY. **(A)** Comparison of RDF profiles of water oxygen atoms around AP*_L_* of MexB (red solid line) and MexY (brown dash-dotted line) extracted from the equilibrium MD trajectories. **(B)** Comparison of SDF of waters within the AP*_L_*. The SDF was calculated over the configurations forming the most populated cluster of MexB (Left) and MexY (Right). The isosurfaces are shown at density isovalue of 2.5 (transparent surface) and 5 (solid mesh). The AP and DP are marked in green and red, respectively, while the G-loop in yellow cartoon representations. The hydrophobic/aromatic residues of the pocket are shown as cyan and orange sticks in the respective structures.

The radial distribution function (RDF) and spatial distribution function (SDF) profiles of water oxygen atoms around the AP*_L_* residues in MexB and MexY were assessed for any possible difference in the density of hydration. The first solvation shell was found at around 2 Å from any residue lining the pocket in both proteins, displaying a slightly lower intensity in MexB (**Figure 5A**). The SDF was calculated on the trajectory of the most populated cluster extracted from MD simulations of MexB and MexY to get more insights into the spatial distribution of water in the pocket (**Figure 5B**). The SDF profiles featured no water density spots near the hydrophobic residues in AP*_L_* of both MexB and MexY but showed a slightly higher number of dense regions in MexY at identical density isovalues (**Figure 5B**). The other lesser populated clusters showed no water dense regions at similar isovalue in both proteins.

From visual inspection, the RDF plots of MexB and MexY are comparable to those of AcrB and AcrD, respectively, which can be related to the similarity in the overall physicochemical makeup of their putative binding pockets. However, MexB featured much less water dense regions than MexY at density isovalues corresponding to the spots found earlier in AcrB and AcrD (Ramaswamy et al., 2017b).

### Deep Pocket of the *Tight* Protomer

The DP was earlier suggested to be the recognition site for low molecular mass compounds and inhibitors (the latter interacting strongly with the HP-trap within this site) (Murakami et al., 2006; Nakashima et al., 2011, 2013; Eicher et al., 2012; Vargiu and Nikaido, 2012; Vargiu et al., 2014; Sjuts et al., 2016; Wang et al., 2017). According to the available X-ray structures of AcrB (Murakami et al., 2006; Seeger et al., 2006; Sennhauser et al., 2007; Eicher et al., 2012) and MexB (Sennhauser et al., 2009; Nakashima et al., 2013), this pocket is open only in the *Tight* protomer; therefore, all the analyses concerning this site were performed on the *Tight* protomer of MexB and MexY.

#### Pocket Volume and Essential Dynamics

**FIGURE 6:**
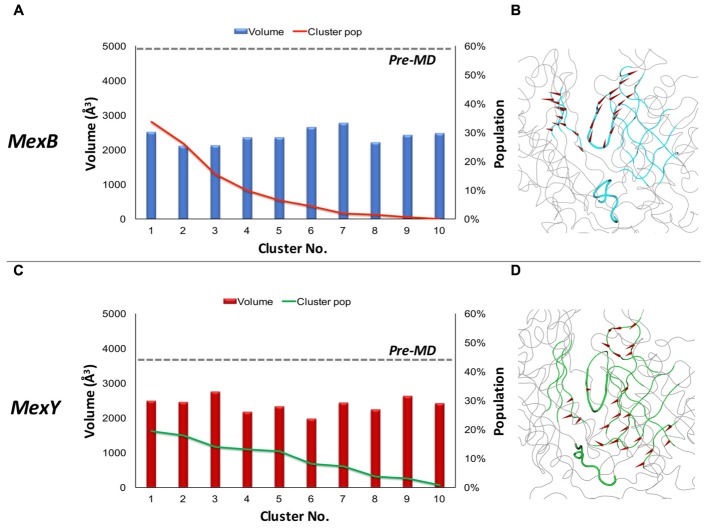
Volume dynamics of DP_T_ of MexB and MexY. (Left panel) Volume distribution of DP*_T_* of MexB **(A)** and MexY **(C)**. (Right panel) Porcupine plots of the PC eigenvector for DP*_T_* of MexB **(B)** and MexY **(D)** simulations shown as arrows (>2Å) attached to Cα atoms indicating the magnitude of the corresponding eigenvalues. The DP*_T_* is highlighted in cyan and green in MexB and MexY, respectively. The G-loop and bottom-loop are shown as thicker tubes.

The volume of the DP*_T_* ranged from 2000 to 2800 Å^3^ in both proteins (average values around 2310 and 2400 Å^3^ for MexB and MexY, respectively), and also the most populated clusters featured a very similar volume of ∼2500 Å^3^ (**Figures 6A,C**). The pre-MD structures showed a much larger DP*_T_* in both proteins (5120 and 3840 Å^3^ in MexB and MexY, respectively) (**Table 2**). This result resembled our previous findings for the major RND transporters of *E. coli* (Ramaswamy et al., 2017b), where the average volumes of the DP*_T_* of AcrB and AcrD were around 2610 and 2770 Å^3^, respectively, during MD, and 3710 and 3850 Å^3^, respectively, in their pre-MD structures. As concluded in that study, despite a large collapse of the pocket (55% in MexB and 37% in MexY) with respect to the conformations in the initial (pre-MD) structures, the DP*_T_* remained large enough to accommodate ligands even in these Mex proteins. A marked dynamical behavior of the DP*_T_* was evident in both transporters as seen from the PCA analysis, the switch-loop and the PN2 (bottom-right region in **Figure 6D**) being the most flexible regions in MexB and MexY, respectively (**Figures 6B,D**).

In MexY, the sterically bulky side chain of W177 (corresponding to F178 in MexB) oriented into the DP*_T_* reduced the volumes in both pre-MD and MD-derived conformations. Also, the populations of the identified clusters indicate a non-preferential distribution of conformations adopted by DP*_T_* in contrast to what we found in Acr transporters (Ramaswamy et al., 2017b), where specific conformations were predominant.

#### Lipophilic Index (LI) and Molecular Lipophilicity Potential (MLP)

**FIGURE 7:**
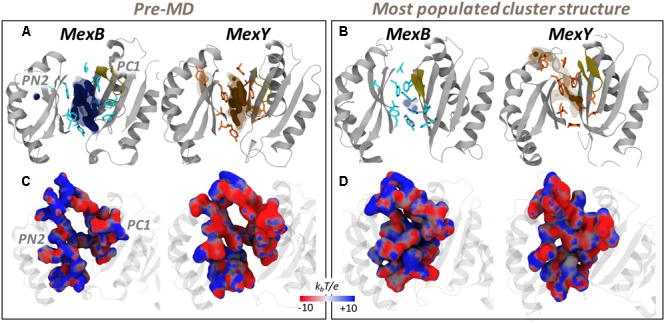
MLP (Upper panel) and electrostatic potential (Lower panel) surfaces of DP*_T_* of MexB and MexY as seen from PC2-PN1 side. **(A,C)** and **(B,D)** correspond to the results from pre-MD and the representative of the most populated cluster, respectively. See **Figure 4** for further details.

The difference between the DP*_T_* of MexB and MexY became more noticeable from their MLP surfaces (**Figures 7A,B**) and LI values (**Table 3**). With its phenylalanine-rich hydrophobic region wide open in MexB, the MLP features high-value isosurfaces over the whole bottom of the DP*_T_*; interestingly, MexY also features a relatively wide and strong MLP in the same region. This result is consistent with the observed higher LI in the pre-MD structure of MexB compared to that of MexY. The differences observed in **Figures 7A,B** and **Table 3** are less marked as compared to that seen for AcrB and AcrD (Ramaswamy et al., 2017b).

An evident reduction in the MLP isosurfaces and in the LI values (for the latter, 80 and 44% in MexB and MexY, respectively) was observed when considering the weighted average computed for the cluster representatives extracted from MD trajectories. This is partly attributed to the larger shrinkage of the DP*_T_* in MexB (55% in volume) than in MexY (37%) thereby influencing the calculation of the LIs as shown for AP*_L_* (see **Table 3** and Supplementary Figure 7).

In the case of DP*_T_*, even though the corresponding HP-trap region is conserved in its overall hydrophobic nature, the residues W177 and Y605 in MexY are less hydrophobic than their phenylalanine counterparts in MexB. Nevertheless, the values of the LI as well as their difference between MexB and MexY are greater in the lesser-conserved DP*_T_* than in the AP*_L_*, as observed for the homologous Acr transporters (Ramaswamy et al., 2017b). This suggests that the DP*_T_* might act even in this case as a lipophilicity-based selectivity filter.

#### Electrostatic Potential

The differences in the electrostatic potential between the DP*_T_* of MexB and MexY appear to be strikingly distinctive like that of the lipophilic potential in these Mex transporters. The electrostatic potential projected onto the surfaces of the DP*_T_* indicated a significantly greater positively charged environment in MexB compared to the more negative pocket of MexY (**Figures 7C,D**). This is consistent with the sequence analysis showing that the DP*_T_* of MexY and MexB are, respectively, composed of around 14 and 5% (2 and 7%) negatively (positively) charged residues. Moreover, these electrostatic features are in good agreement with the desired complementarity needed to accommodate the charged substrates transported by these proteins. The greater negative charge in the DP*_T_* of MexY favors positively charged aminoglycosides and disfavors negatively charged molecules; however, along with the scattered positive charges, the DP*_T_* in MexY may feebly favor binding of β-lactams (especially zwitterionic). Likewise, MexB with its predominant positive electrostatic potential surface in the DP*_T_* may attract negatively charged as well as zwitterionic β-lactams, and extrude them with greater efficiency along with weakly acidic quinolones such as cinoxacin and nalidixic acid, in comparison to its lower efficiency in pumping out cationic antibiotics (oleandomycin, erythromycin, and puromycin) (**Table 1**).

#### Hydration Analysis

**FIGURE 8:**
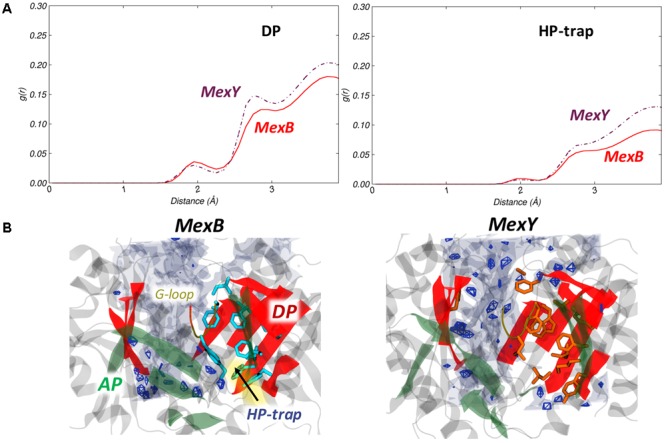
Hydration of DP*_T_* and HP-trap in the *T* protomer of MexB and MexY. **(A)** Comparison of RDF profiles of water oxygen atoms around the DP*_T_* and HP-trap in the *T* protomer of MexB (red solid line) and the corresponding regions of MexY (brown dash-dotted line). **(B)** Comparison of SDF for waters in the DP*_T_* calculated over the configurations forming the most populated cluster of MexB (Left) and MexY (Right) illustrating the variation in the immediate environment of the hydrophobic residues. The position of the HP-trap of MexB is indicated by an arrow. See **Figure 5** for further details.

The overall hydration of the DP*_T_* and HP-trap as reflected by the RDF plot was not very different between the Mex proteins (**Figure 8A**). In contrast, the spatial positions of water dense regions as seen from the SDF (**Figure 8B**) showed the DP*_T_* of MexY with more high-density regions than that of MexB, possibly due to a greater number of charged residues in the translocation channel part of the DP*_T_*. The HP-trap region was devoid of water in both proteins due to the shrinkage of the pockets during MD. The presence of a polar residue (Y605) might have had a minor influence on the hydration of the corresponding HP-trap region in MexY, provided it was less buried by the hydrophobic bulky side chain of W177.

### Fragment-Based Binding Site Characterization

MDR transporters like the Mex pumps investigated here often feature recognition sites endowed with several binding hotspots (Vargiu and Nikaido, 2012; Ruggerone et al., 2013; Yamaguchi et al., 2015) whose number, strength and spatial distribution determine the level of promiscuity of their interactions (Ciulli et al., 2006). Therefore, by using fragment moieties (Supplementary Figure 8) characterized by different physicochemical features, we probed the AP*_L_* and DP*_T_* of the two proteins to map their possible MFSs (Imai et al., 2011; Ramaswamy et al., 2017b).

**FIGURE 9:**
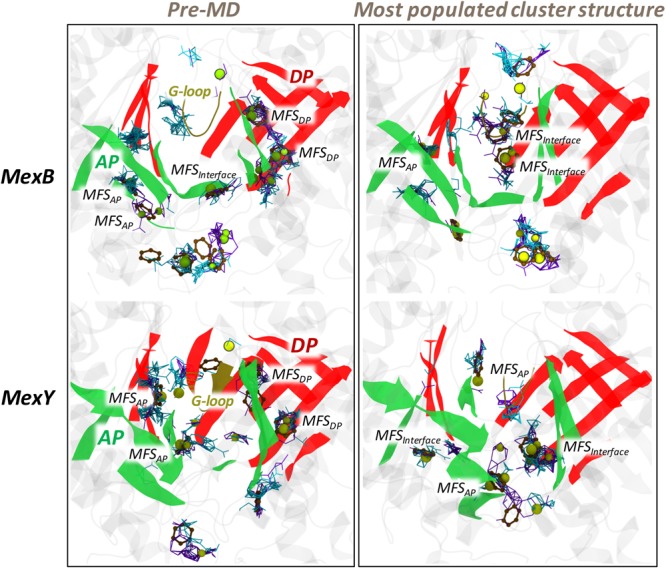
The various MFSs identified in the AP*_L_* and DP*_T_* of MexB and MexY. The binding modes of the different probes are shown as lines for hydrogen-bond donor (cyan) and hydrogen-bond acceptor (violet), as beads for aliphatic (yellow), and as CPK for aromatic (ochre) ligands. The AP and DP are marked in green and red, respectively, while the G-loop in yellow cartoon representations. (Note: The categorizing of MFSs here is arbitrary due to indistinct boundaries between the pockets. The sites not labeled as MFS here are all CSs; for further details see section “Materials and Methods”).

As expected, several MFSs were identified within the AP*_L_* and DP*_T_* of both transporters (**Figure 9** and Supplementary Table 3). In particular, MexY had a larger (lower) number of MFSs in the AP*_L_* (DP*_T_*) than MexB. Considering the pre-MD and the top 5 clusters extracted from MD trajectories, the AP*_L_* and the interface/G-loop region almost always showed the presence of at least 1 MFS in both proteins. For the DP*_T_*, however, a marginal difference was found, with MexB housing an average of 1 MFS compared to 0.7 in MexY. This difference was greater in the DP*_T_* of Acr transporters (AcrB with 1.3 and AcrD with 0.3 MFSs on average) (Ramaswamy et al., 2017b), which feature greater diversity in their substrate profile as compared to MexB and MexY. Note that the MFS identified in the DP*_T_* of the pre-MD structure in MexB is located exactly where the inhibitor D13-9001 (Nakashima et al., 2013) was experimentally resolved. In comparison to AcrB crystal structures, this is the site where several substrates like minocycline (Murakami et al., 2006; Nakashima et al., 2011; Eicher et al., 2012), doxorubicin (Murakami et al., 2006; Eicher et al., 2012), and inhibitors like D13-9001 (Nakashima et al., 2013) and MBX compounds (Sjuts et al., 2016) were resolved. Conformational changes during the MD simulation impacted the number and location of MFS compared to their pre-MD structures, nevertheless retaining the promiscuity in both transporters (Supplementary Figure 9 and Supplementary Table 3). An interesting feature was that though several consensus sites (CSs) populated with hydrogen bond donors and acceptors were observable in both proteins, those of MexB had many more aromatic-preferred sites than MexY.

The position of the MFSs was not the same for all MD-derived clusters (Supplementary Figure 9 and Supplementary Table 3) and this dynamicity (in addition to the scattered profile) in the distribution of MFSs results from the exposure of different weak binding sites during conformational changes in the protein. Presence of such MFSs is likely very important to avoid the substrate from being trapped in a single site and to facilitate its efflux by multisite-drug-oscillation (Yamaguchi et al., 2015).

## Conclusion and Perspectives

We presented here an extensive comparative investigation of the structural and dynamic features of the two major RND multidrug transporters in *P. aeruginosa*, MexB and MexY. To the best of our knowledge this is the first structure-based study of MexY and also the first thorough quantitative comparison of the main putative binding pockets of the two transporters. We identified specific features of their multidrug binding pockets that partly explain the similarities and differences in their substrate selectivity profiles. Both proteins feature dispersed (mosaic-like) profiles of lipophilic and electrostatic surfaces within their access and deep binding pockets, which provide several multifunctional sites for diffuse binding of chemically dissimilar compounds. Several differences spotted in the molecular descriptors of the binding sites of MexB and MexY can be related to their different specificity profiles. Our results point out that the lesser conserved DP*_T_* could likely be the major substrate selection site in both proteins. In addition, the observed dynamics of the bottom-loop support our earlier hypothesis for Acr pumps of *E. coli* (Ramaswamy et al., 2017b) that their different dynamics contributes to the binding of substrates of different sizes. Collectively, our findings add a valuable piece to fill in the knowledge gap in molecular recognition and transport by bacterial RND transporters, an issue of importance in addressing antibiotic resistance.

## Materials and Methods

The protocol followed in this study is the same as that we used in our previous work for Acr transporters of *E. coli* (Ramaswamy et al., 2017b).

### Homology Modeling of MexY

Since no experimental structure of MexY has been solved yet, we built a model of its asymmetric trimer structure by multiple template-based homology modeling using Modeler 9.13 (Šali and Blundell, 1993). The amino acid sequence of full length MexY transporter protein from *P. aeruginosa* PAO1 was retrieved from the UniProt database (The UniProt Consortium, 2015) (UNIPROT ID: Q9ZNG8), and subsequently searched for the best available template structures bearing homologous relationship to the query sequence using the NCBI-BLAST tool (Madden, 2013) against the Protein Data Bank (PDB) ^2^. The high-resolution crystal structure of AcrB at 1.9 Å [PDB code 4DX5 (Eicher et al., 2012)] and MexB at 2.7 Å [PDB code 3W9I (Nakashima et al., 2013)] were chosen as templates for multiple-template based modeling of MexY. The protein sequences were optimally aligned by ClustalOmega (Sievers et al., 2011) and the results were visually inspected to ensure the absence of gaps in important secondary structure regions. Modeler 9.13 (Šali and Blundell, 1993) was used to generate a total of 100 asymmetric models of MexY based on AcrB and MexB templates using an optimization method combining slow MD with very thorough variable target function method through 300 iterations, and this whole cycle was repeated twice unless the objective function MOLPDF was greater than 10^6^. The resulting models were ranked using discrete optimized protein energy (DOPE) (Shen and Sali, 2006) score values, and the top 5 models (with the lowest DOPE score) were selected for individual structure quality checks. Each model was further subjected to loop refinement using Modeler, and to overall structure relaxation by energy minimizations using AMBER14 (Case et al., 2014). The most reliable model was then selected based on various geometric and stereochemical quality factors evaluated for backbone angles, side chains flips, rotamers, steric clashes etc. using PROCHECK (Laskowski et al., 1993), ERRAT (Colovos and Yeates, 1993), ProSA (Wiederstein and Sippl, 2007), Verify3D (Eisenberg et al., 1997) programs available in MolProbity (Chen et al., 2010) and Structure Analysis and Verification Server^3^.

We performed comparative structural evaluation by superimposition of the modeled MexY structures over experimentally determined X-ray crystal structures of AcrB and MexB used as templates. Likewise, the template structures were also evaluated with the same programs to serve as reference for the results obtained for the MexY models. Visual inspections were performed with VMD1.9.1 (Humphrey et al., 1996) and PyMOL (Schrödinger, 2015).

### Molecular Dynamics Simulations of MexB and MexY

Molecular dynamics simulations of the crystal structure of MexB (PDB code 3W9I) and of the most reliable homology model of MexY were carried out using the AMBER14 (Case et al., 2014) program. Protomer specific protonation states (Eicher et al., 2014) were adopted with E346 (E345) and D923 (D919) protonated in both *Loose* and *Tight* protomers while deprotonated in the *Open* protomer of MexB (MexY). The residues D407 (D406), D408 (D407), D566 (E563) were protonated only in the *Open* protomer of MexB (MexY). The proteins were successively embedded in 1-palmitoyl-2-oleoyl-sn-glycero-3-phosphoethanolamine (POPE) bilayer patches, solvated with explicit TIP3P water model. The residual charge of the systems was neutralized by appropriate numbers of randomly placed K^+^/Cl^-^ ions (Schulz et al., 2010, 2011, 2015; Vargiu et al., 2011). The ions count was suitably adjusted to account for an osmolarity of 0.15 M KCl. Embedding of the protein into a pre-equilibrated POPE bilayer patch was done using the PPM server (Lomize et al., 2012) and subsequently the CharmmGUI tool (Jo et al., 2008). The lipid residue nomenclature was converted from the CHARMM to AMBER format using the *charmmlipid2amber.py* python script provided with AmberTools. The central pore lipids were added after calculating the number of lipids to be added to each leaflet by dividing the approximate area of the central pore by the standard area per lipid of POPE molecules (Dickson et al., 2014). The topology and the initial coordinate files were created using the *LEaP* module of AmberTools14. Periodic boundary conditions were used and the distance between the protein and the edge of the box was set to be at least 30 Å in each direction.

Multi-step energy minimization with a combination of steepest descent and conjugate gradient methods was carried out using the *pmemd* program implemented in AMBER14 to relax internal constrains of the systems by gradually releasing positional restraints. Following this, the systems were heated from 0 to 310 K by a 1 ns heating (0–100 K) under constant volume (NVT) followed by 5 ns of constant pressure heating (NPT) (100–310 K) with the phosphorous heads of lipids restrained along the *z*-axis to allow membrane merging and to bring the atmospheric pressure of the system to 1 bar. Langevin thermostat (collision frequency of 1 ps^-1^) was used to maintain a constant temperature, and multiple short equilibration steps of 500 ps under anisotropic pressure scaling (Berendsen barostat) in NPT conditions were performed to equilibrate the box dimensions. A time step of 2 fs was used during all these runs, while post-equilibrium MD simulations were performed with a time step of 4 fs under constant volume conditions after hydrogen mass repartitioning (Hopkins et al., 2015). The particle-mesh Ewald (PME) algorithm was used to evaluate long-range electrostatic forces with a non-bonded cutoff of 9 Å. During the MD simulations, the length of all R-H bonds was constrained with SHAKE algorithm. Coordinates were saved every 100 ps. The ff14SB (Maier et al., 2015) version of the all-atom Amber force field was used to represent the protein systems while lipid14 (Dickson et al., 2014) parameters were used for the POPE bilayer. After equilibration, multi-copy μs-long MD simulations were performed for each system, namely four ∼1 μs-long production simulations for each transporter (for a total simulation time of ∼8 μs). Trajectory analysis was done using *cpptraj* module of AmberTools14 and VMD1.9.1, and graphs were plotted using the *xmgrace* tool.

### Principal Component Analysis

To characterize and highlight possible similarities and differences in the collective motions of the binding pockets, we calculated the covariance matrices from the equilibrium trajectory and performed a PCA (García, 1992; Daidone and Amadei, 2012). As customary in PCA analysis, the covariance matrix was constructed taking the three-dimensional positional fluctuations of Cα atoms from their ensemble average position (after least-squares fitting to remove rotational and translational motion). Diagonalization of the covariance matrix yields a set of eigenvectors and corresponding eigenvalues, which represent the direction and amplitude of the motion, respectively. The eigenvectors are then ranked according to the decreasing order of their associated eigenvalues, such that the first eigenvector represents the largest contribution to the total fluctuation of the system. To visualize the motions represented by the eigenvectors, the structures from the trajectories can be projected onto each eigenvector of interest [principal component (PC)] and transformed back into Cartesian coordinates. The two extreme projections along each eigenvector can then be interpolated to create an animation or compared to understand which parts of the protein are moving according to that specific eigenvector and to what extent. Usually (a combination of), the first few principal components are able to represent most of the collective motions [the “essential dynamics” (Daidone and Amadei, 2012)] occurring in an MD simulation among the different regions of a protein.

### Clustering of MD Trajectories

A cluster analysis of the MD trajectories was performed using the average-linkage hierarchical agglomerative clustering method implemented in *cpptraj* module of AMBER. Such clustering helps to reduce the number of structures for analysis yet retaining the large conformational space sampled during the MD runs. In this approach, we clustered in two separate instances the trajectory based on root mean square deviation (RMSD) (cutoff set to 3 Å) of the AP in *L* protomer and of the DP in *T* protomer. For each protein, the representative structures from each of the 10 top clusters generated in each of the two cases considered (AP in *L*, DP in *T*) were used to perform quantitative analyses in order to account for dynamical behavior. All non-protein molecules were stripped from the trajectory during post-processing to reduce additional memory usage and to speed up file processing.

### Pocket Descriptors

The list of the pocket descriptors identified for the present study includes: (i) cavity volume; (ii) molecular lipophilicity potential; (iii) electrostatic potential; (iv) site hydration; and (v) fragment-based binding site characterization. The various pocket descriptors used to characterize the binding site were calculated using specific programs after validating their applicability to RND systems by assessing results against available crystal structures and experimental data, as well as previous computational reports (Imai et al., 2011; Schulz et al., 2011; Vargiu et al., 2011, 2014; Vargiu and Nikaido, 2012; Fischer and Kandt, 2013; Ramaswamy et al., 2017b).

#### Cavity Volume

Evolution of size and shape of the AP and DP during MD simulations was examined using the two-probe sphere method of *rbcavity* program bundled in the rDock suite (Ruiz-Carmona et al., 2014). This allows obtaining detailed information on the pocket volume and plasticity of the site. In this method, the binding site volume was identified by a fast grid-based cavity detection algorithm (Morley and Afshar, 2004) within a sphere of radius 13 Å for AP*_L_* and 14 Å for DP*_T_*, centered over the pockets, using large and small probe radii of 6.0 and 1.5 Å, respectively. These radii were found to be optimal for our case after evaluating different combinations and checking through visual inspection their accuracy in predicting volume of the pocket space by keeping the possible inclusion of regions extending outside the pocket of interest at its least.

#### Molecular Lipophilicity Potential

The three-dimensional distribution of lipophilicity in space or on a molecular surface can be described using molecular lipophilicity potential (MLP), which represents the influence of all lipophilic fragmental contributions of a molecule on its environment. The MLP value of a point in space (*k*) is generated as the result of intermolecular interactions between all fragments in the molecule and the solvent system, at that given point. Thus, MLP can be calculated from the fragmental system of logP and a distance function as shown in the following equation (Gaillard et al., 1994):

$$\begin{array}{c} {\mathrm{MLP}}_{k}\;=\;\;\overset{N}{\underset{i=1}{\Sigma }}F_{i}.f(d_{ik}) \end{array}$$

where *N* is the number of fragments, *F_i_* is the lipophilic contribution of fragment *i* of the molecule and *f(d_ik_)* is a function based on the distance of the measured point in space *k* to fragment *i*.

In this way, summing up all positive and all negative MLP values associated to each point on the binding pocket yields the lipophilic index (LI) as:

$$\begin{array}{c} \mathrm{LI}\;\;=\;\frac{\Sigma {\mathrm{MLP}}^{+}}{\Sigma {\mathrm{MLP}}^{+}+|\;\Sigma {\mathrm{MLP}}^{-}\;|}\;.\;100 \end{array}$$

The lipophilicity of AP in *L* protomer and of DP in *T* protomer were qualitatively and quantitatively estimated in this way using MLP Tools (Oberhauser et al., 2014) plugin available for PyMOL.

#### Electrostatic Potential

The electrostatic potential surface maps were computed by APBS (Baker et al., 2001), after pre-processing structures of MexB and MexY to assign charges and atomic radii using the PDB2PQR server (Dolinsky et al., 2004). All electrostatic potential calculations were performed at 0.15 M physiological salt concentration, with a solvent probe of radius 1.4 Å, a solvent dielectric constant of 78.5, a biomolecular dielectric constant of 2.0, a temperature of 310 K, a minimum grid spacing of 0.5 Å and keeping the other Poisson–Boltzmann parameters at default.

#### Hydration Analysis

The RDF indicates the probability of finding water molecules at a certain distance from a region or residue of interest and is commonly used to analyse the solution structure revealed from either experimental or computer simulations data.

The RDF analysis of water oxygen atoms was performed using *cpptraj* module of AMBER14, in which the RDF is computed from the histogram of the number of solvent particles found as a function of the distance *R* from an (ensemble of) atom(s), normalized by the expected number of solvent particles at that distance in bulk. The normalization is estimated from:

$$\begin{array}{c} Density^{*}\;\left(\left[\frac{4\pi }{3}{\left(R+dR\right)}^{3}\right]-\;\left[\frac{4\pi }{3}dR^{3}\right]\right) \end{array}$$

where *dR* is equal to the bin spacing, the default *density* value is 0.033456 molecules Å^-3^, which corresponds to density of water approximately equal to 1.0 g mL^-1^. Bin spacing of 0.1 and a maximum bin value of 4.0 was used in this case to calculate the RDF of all water oxygen atoms to each atom of AP in *L* protomer and of DP in *T* protomer over the entire length of the simulation.

Though RDF clearly shows a difference in the water distribution around the desired regions, it lacks the ability to present the information about the spatial positions of these differences. Hence, SDF of waters around the whole protein was calculated using the Gromacs utility *g_spatial* (Abraham et al., 2015) on the trajectory frames grouped into the most populated conformational clusters extracted from MD simulations. SDF allows determining the three-dimensional density distribution of aqueous solution around the binding pockets of the transporters. RDF and SDF together highlight the hydration around the binding pockets of these proteins, which can be effectively used to understand the molecular mechanism of interaction of water molecules penetrating the pocket in a dynamic manner.

#### Fragment-Based Binding Site Characterization

The FTMap server (Kozakov et al., 2015) implementing the FTSite algorithm is a tool helpful in the identification of binding sites and of the fragments that could be possible source of structure- and fragment-based drug design attempts. The main aim of such fragment-based binding site analysis is to obtain a measure of the ability of the protein (and in particular the pockets under study) to bind a drug-like molecule.

FTMap identifies the important hot spots based on the consensus clusters of 16 standard probes which include molecules varying in size, shape and polarity (Supplementary Figure 8). Such a diverse library of probes is useful to capture a range of interaction types that include hydrophilic, hydrophobic, hydrogen-bonding and aromatic interactions. The regions where clusters of different probes of the same or different type overlap are marked as CSs and MFSs, respectively, and are ranked based on the number of their clusters. Clusters in close proximity to a top ranked cluster are merged with it and the protein residues within this region become the top ranked putative ligand binding site.

## Author Contributions

VR performed homology modeling, MD simulations and analysis. VR, AV, GM, and PR analyzed the results. VR, AV, GM, JD, and PR designed the experiments, discussed the results, and wrote the manuscript. All authors contributed to manuscript revision, read and approved the submitted version.

## Conflict of Interest Statement

The authors declare that the research was conducted in the absence of any commercial or financial relationships that could be construed as a potential conflict of interest.

## Footnotes

1 http://www.uniprot.org/blast

2 www.rcsb.org

3 http://services.mbi.ucla.edu/SAVES/
