# Supplementary material for: Molecular Determinants of the Promiscuity of MexB and MexY Multidrug Transporters of Pseudomonas aeruginosa
Source: Front Microbiol. 2018 Jun 1;9:1144. doi: 10.3389/fmicb.2018.01144 (PMC5992780; doi:10.3389/fmicb.2018.01144)
Supplement: Supplementary file 1 [file Data_Sheet_1.pdf]

Supplementary Material

Molecular determinants of the promiscuity of MexB and MexY multidrug transporters of *Pseudomonas aeruginosa*

Venkata Krishnan Ramaswamy[1], Attilio V. Vargiu[1], Giuliano Malloci[1], Jürg Dreier[2], Paolo Ruggerone[1*]

[1] Department of Physics, University of Cagliari, Cittadella Universitaria, Monserrato (CA), Italy
[2] Basilea Pharmaceutica International Ltd., Basel, Switzerland

*** Correspondence:** Paolo Ruggerone: paolo.ruggerone@dsf.unica.it

1 Supplementary Data

Homology modeling of MexY

Both MexB of *P. aeruginosa* and AcrB of *E. coli* sequences showed a comparable identity (~47% and ~48%, respectively) and similarity (~66% and ~67%, respectively) with MexY sequence of *P. aeruginosa* with least gaps (none in the Access and Deep binding pockets) over maximum sequence coverage. All data are collected in the Supplementary Table 1. Visual inspection of the top 5 homology models of MexY generated with MODELLER by multiple template-based homology modeling revealed an overall identical 3D structural fold characteristic of RND transporters. The final MexY model featured 94.3% and 99.2% of residues in the favored and allowed regions of the Ramachandran plot, respectively, and the evaluation results were fully within the permissible limits of a good model. The overall quality factor for non-bonded atomic interactions assessed with ERRAT plot further confirmed the high quality of our model with a score of 93.34%, which was in the range of high-resolution experimental structures. The ProSA evaluation showed that the overall model quality (Z-score) of the homology model (-11.6) and the template crystal structures (-12.4; -12.3) were comparable and within the range of scores typically found for native proteins of similar size. The local quality of the model based on interaction energies for each residue as evaluated with ProSA served as an additional verification of model quality with most of the residues falling in the negative energy scale and only a minor set on the positive side.

The evaluation results are summarized in Supplementary Table 2, which also shows the structure of the MexY model superposed to its AcrB (PDB code 4DX5) and MexB (PDB code 3W9I) template structures, highlighting the similarity in their general structural fold. Note that in the MexB crystal structure, the PC2 subdomain is significantly shifted towards the transmembrane domain and towards PC1, which was also discussed by Sennhauser *et al.* based on their first published X-ray structure of MexB (Sennhauser et al., 2009). This conformational shift constricted the substrate entrance channel towards the periplasmic side formed by the PC domains in MexB. This channel between PC1 and PC2 is open in AcrB, continuing to the access pocket in the *Loose* and *Tight* protomers. The MexY model chosen in our case resembles AcrB conformation in this aspect.

All the model assessment results were within ranges observed for the experimentally (X-ray) determined template structures (PDB codes 4DX5 and 3W9I), increasing the confidence in the MexY homology model obtained.

2 Supplementary Figures and Tables

2.1 Supplementary Figures

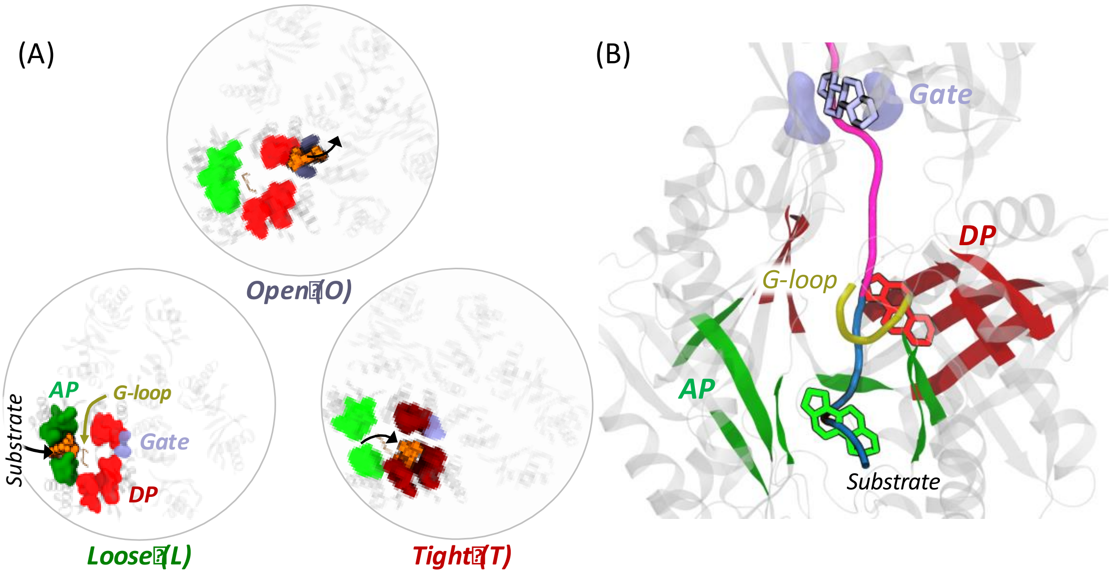

Supplementary Figure 1. (A) Proposed functional rotation mechanism and (B) putative substrate transport pathway from the AP to the exit Gate going through the DP in RND transporters [adapted from (Ramaswamy et al., 2017)]. The substrate is shown as orange van der Waals spheres in panel (A) while it is shown in licorice colored differently (green, red or iceblue) depending on its stage along the transport path in panel (B). AP is displayed in green color, DP in red and Gate in iceblue. The thick tube displayed in (B) indicates in iceblue and magenta the path followed by substrates in different stages of the transport cycle.

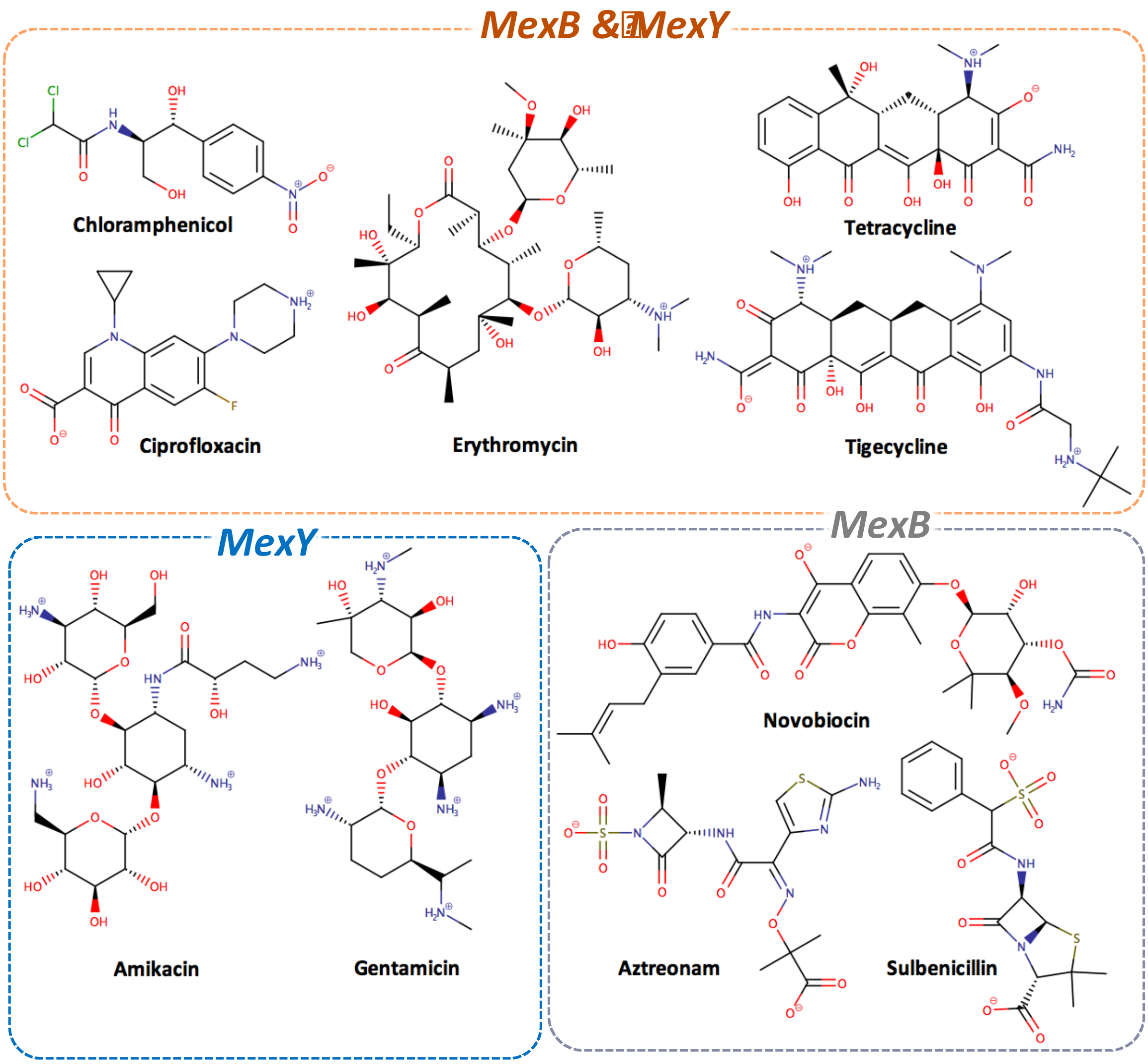

Supplementary Figure 2. Examples of antibiotic substrates of MexB and MexY in *P. aeruginosa*. Grey and blue frames indicate substrates of MexB and MexY, respectively, while the orange frame indicates the substrates common to both transporters.

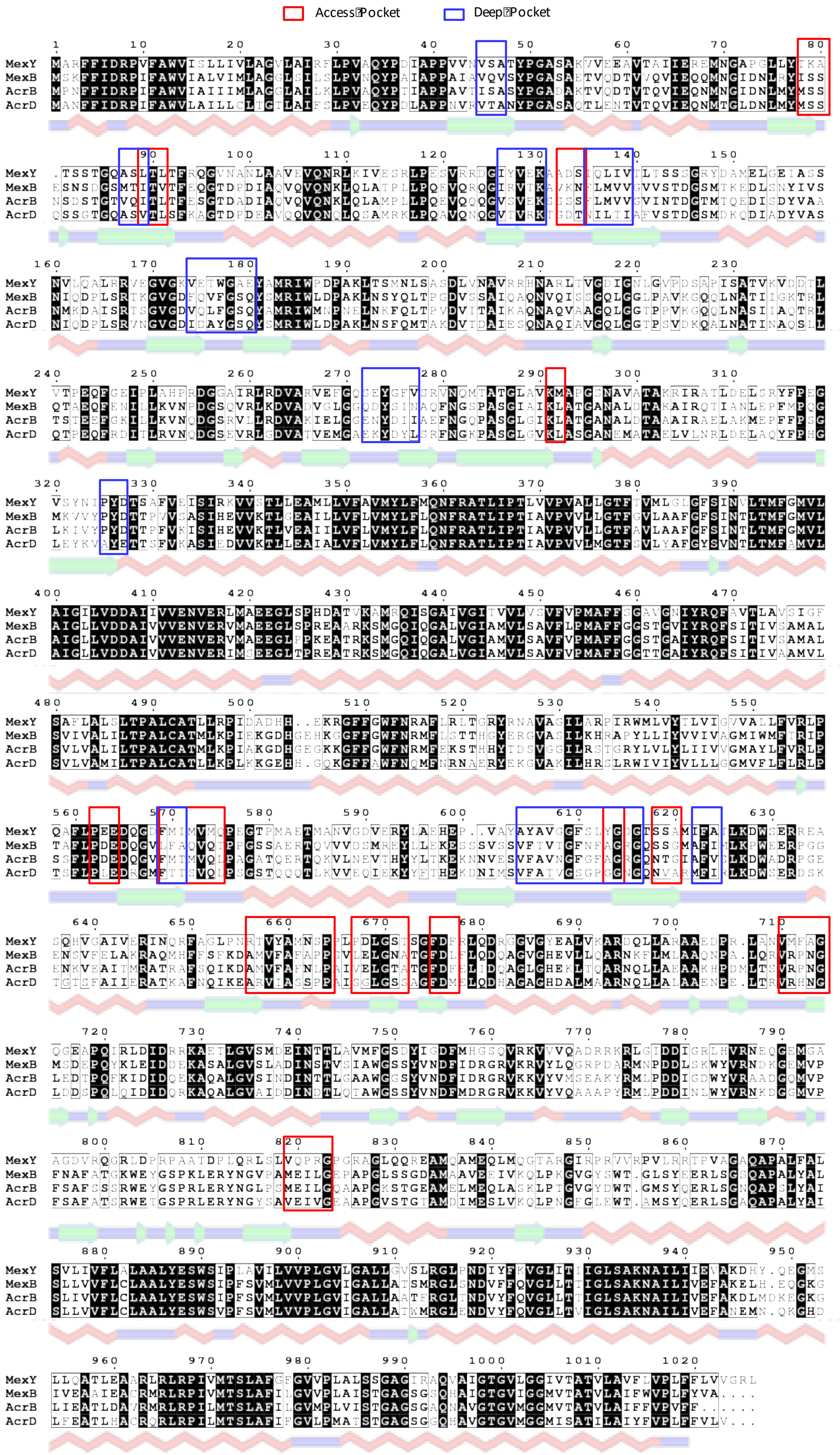

Supplementary Figure 3. Sequence alignment of MexY with MexB and AcrB. The regions corresponding to Access Pocket (AP) and Deep Pocket (DP) are marked in red and blue boxes, respectively. The secondary structure of the sequence is also shown with β-sheet in green, α-helix in red and coil in blue. Identical residues are highlighted with black filled boxes, similar with colorless boxes while all others are mismatches.

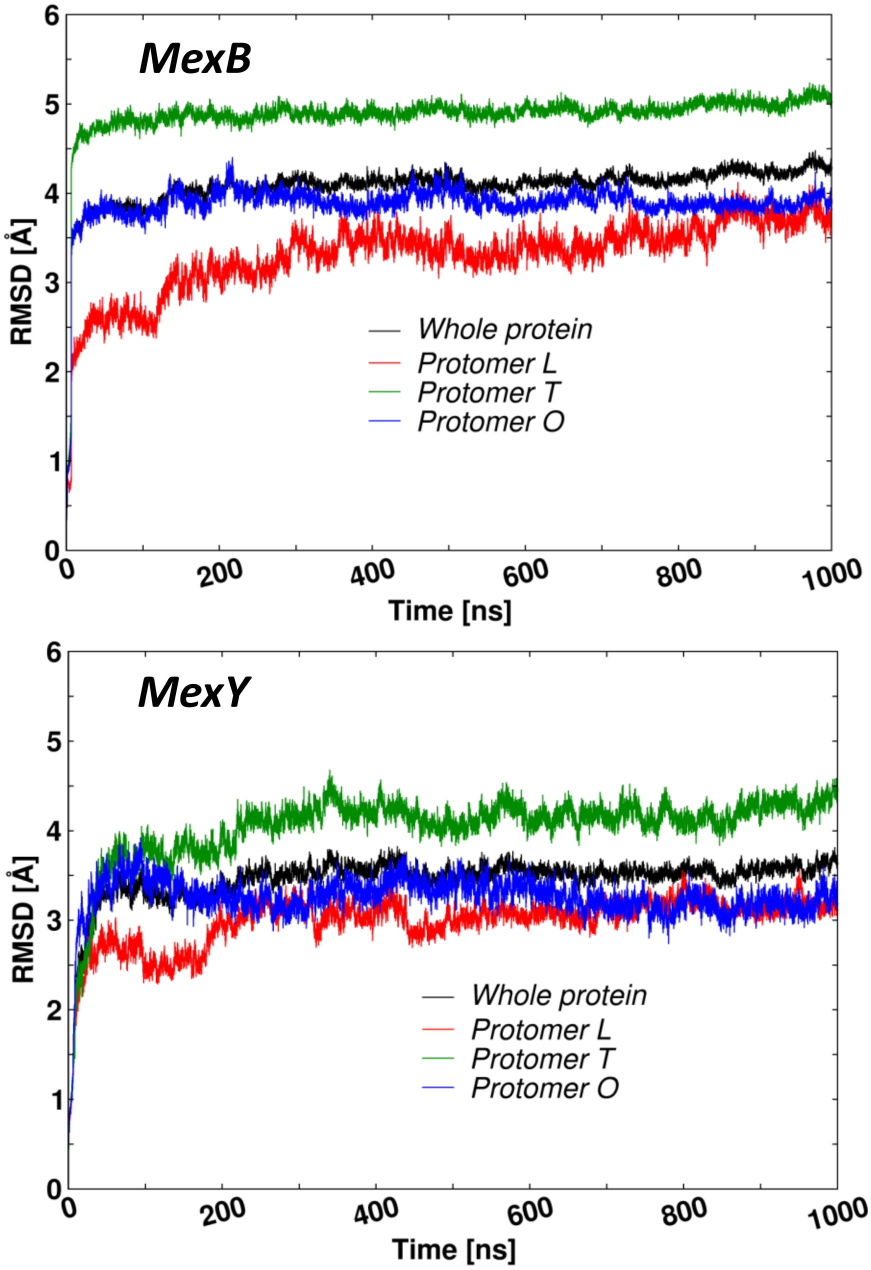

Supplementary Figure 4. RMSD of the backbone of whole proteins and of each protomer for MexB (upper panel) and MexY (lower panel). The RMSD is calculated with respect to the pre-MD reference structure and plotted as a function of the simulation time.

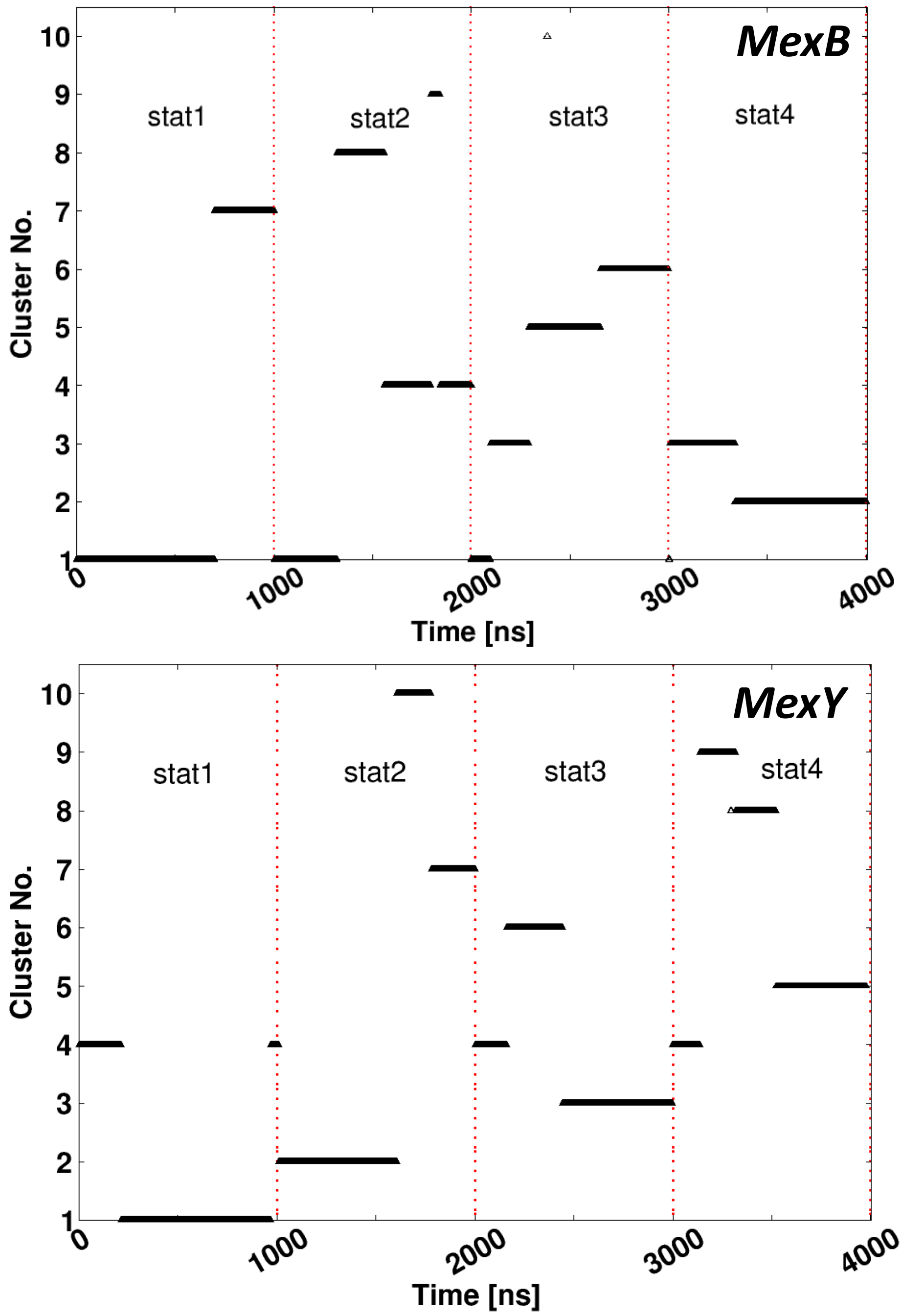

Supplementary Figure 5. Cluster evolution timeline for AP$_L$ of MexB (upper panel) and MexY (lower panel). The red dotted lines mark the corresponding trajectory sections of the multiple MD runs (labelled as statN, N=1, 2, 3, 4).

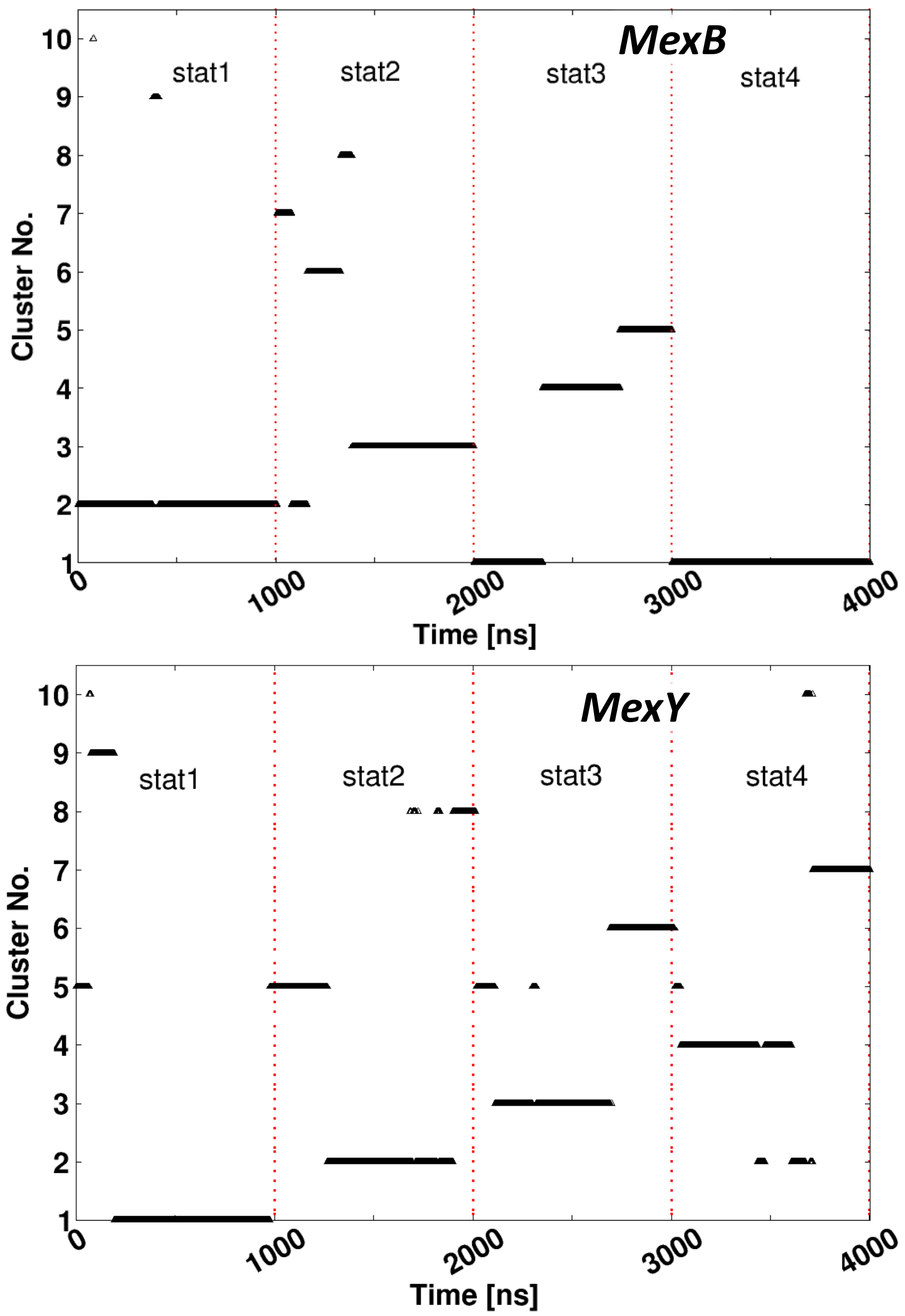

Supplementary Figure 6. Cluster evolution timeline for DP$_T$ of MexB (upper panel) and MexY (lower panel). The red dotted lines mark the corresponding trajectory sections of the multiple MD runs (labelled as statN, N=1, 2, 3, 4).

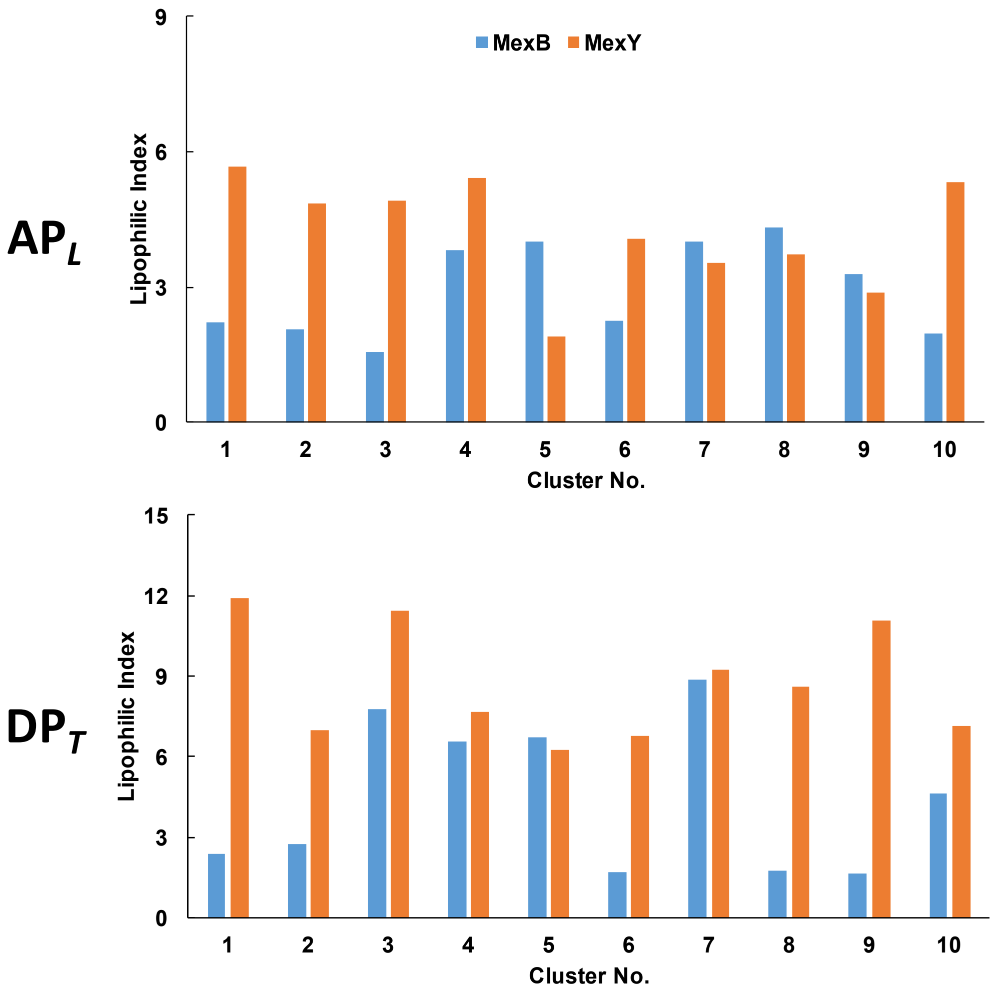

Supplementary Figure 7. Distribution of the lipophilic index for AP_L (upper panel) and DP_T (lower panel) of MexB and MexY over the cluster representatives extracted from equilibrated MD trajectories.

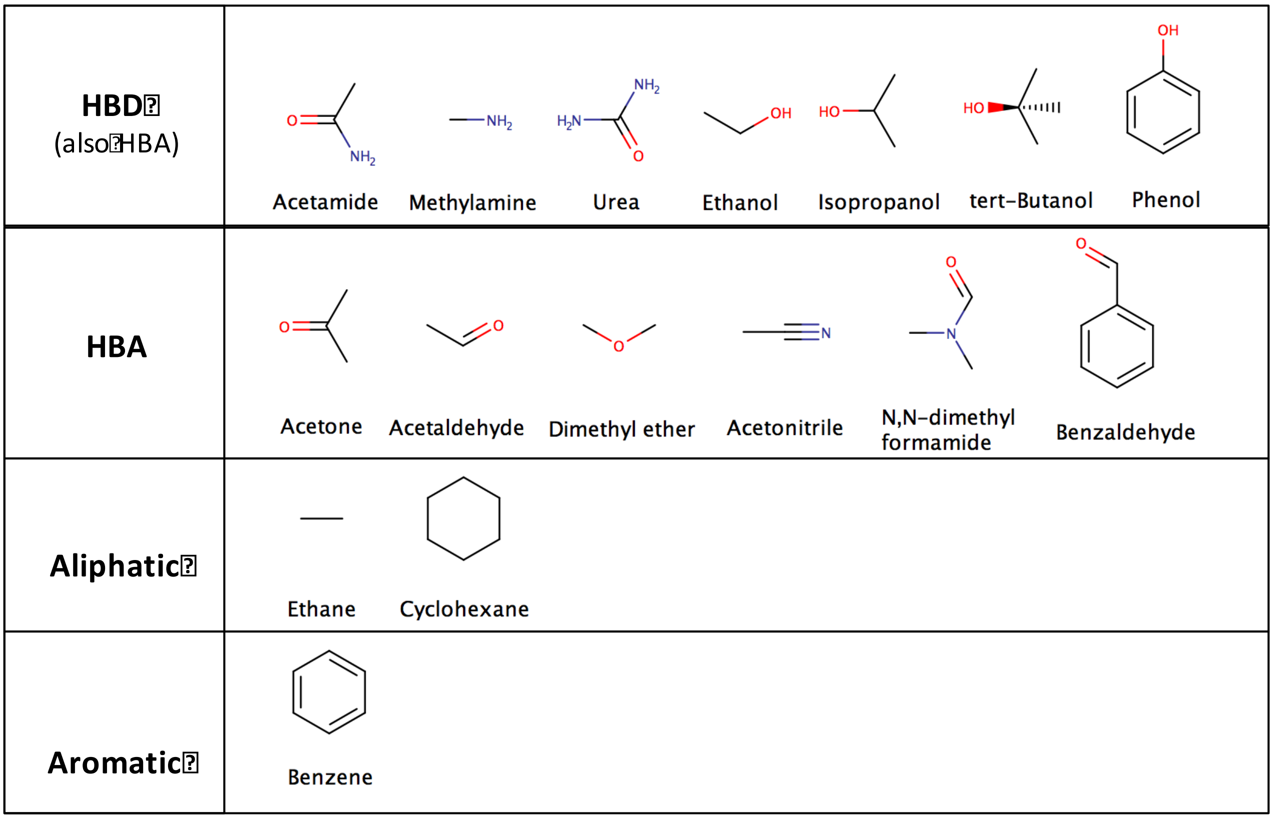

Supplementary Figure 8. The standard repertoire of small organic probe molecules used by FTMap. (HBD: Hydrogen Bond Donors; HBA: Hydrogen Bond Acceptors)

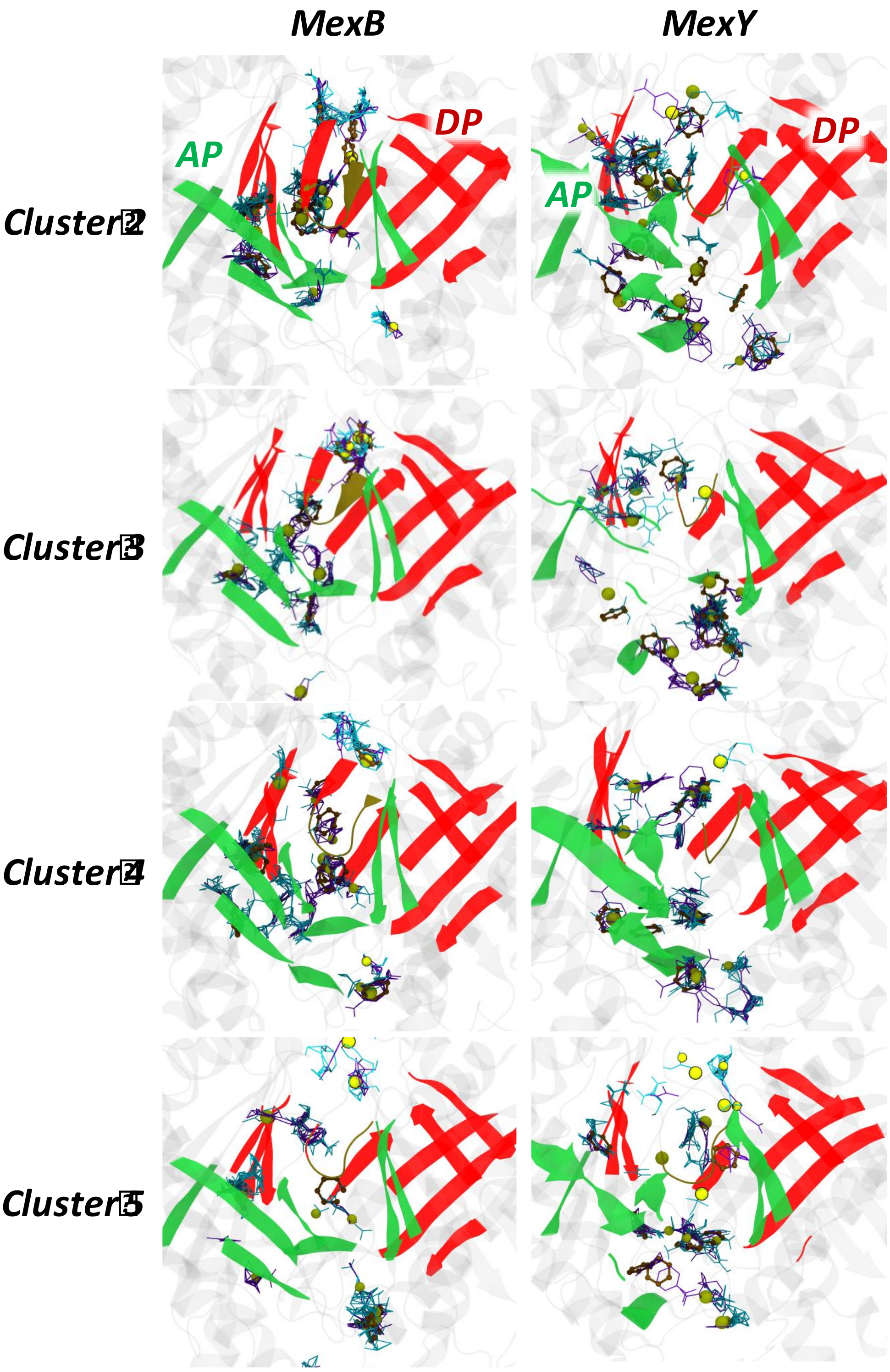

Supplementary Figure 9. The distribution of various MFSs within the AP and DP of top five cluster representatives. Refer to Figure 9 in main text for cluster 1. The binding modes of the different probes are shown as lines for hydrogen-bond donor (cyan) and hydrogen-bond acceptor (violet), as beads for aliphatic (yellow), and as CPK for aromatic (ochre) ligands. The AP and DP are marked in green and red, respectively, while the G-loop in yellow cartoon representations. (Note: The categorizing of MFS here is arbitrary due to indistinct boundaries between the pockets).

2.2 Supplementary Tables

Supplementary Table 1. Sequence identity (similarity) between RND transporters of *E. coli* and *P. aeruginosa* calculated using EMBOSS Stretcher (Rice et al., 2000) (All values are in percentages)

System	MexY	AcrB	AcrD
MexB	46.9 (65.5)	69.8 (83.2)	61.1 (76.5)
MexY	-	47.9 (67.0)	48.2 (66.7)
AcrB	-	-	65.3 (79.7)

Supplementary Table 2. Evaluation results of the multiple-template based MexY model. (Left panel) Structural superposition of MexY (colored domain-wise) onto MexB and AcrB (both in grey in the respective inset). Only a single protomer is shown for the sake of clarity. The table on the right shows the evaluation results for MexY model obtained with templates AcrB (PDB code 4DX5), MexB (PDB code 3W9I) and multi-template.

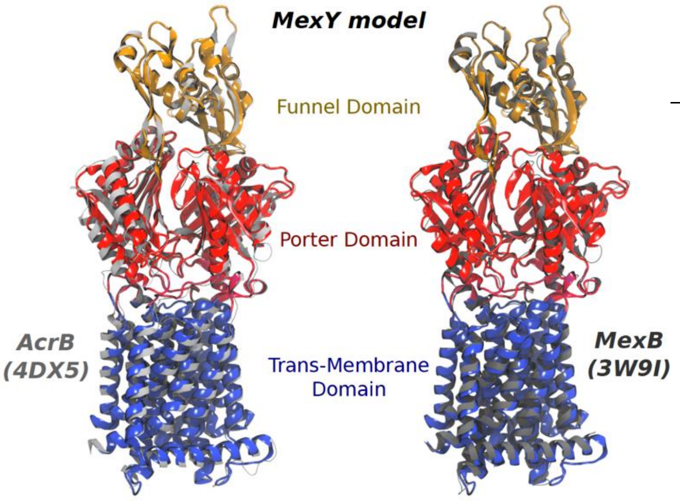

Evaluation Criteria	MexY homology model		
	AcrB	MexB	Multi-template
RMSD* (Cα-atoms)	0.9 Å	1.1 Å	1.2 Å (MexB)<br>1.1 Å (AcrB)
TM-score	0.9	0.9	0.9
Ramachandran favored	95.2%	93%	94.3%
Errat	93.1%	92.9%	93.3%
Verify-3D	Pass	Pass	Pass

*RMSD calculation was done by structural alignment using *cealign* command in PyMOL (Schrödinger, 2015).

Supplementary Table 3. The number of MFSs identified in the binding pockets of MexB and MexY before and during MD.

Structure		Number of MFS			Total number of MFS
		AP	DP	Interface/G-loop	
MexB	Pre-MD	2	2	1	5
	Cluster 1	1	-	2	3
	Cluster 2	1	1	1	3
	Cluster 3	2	2	-	4
	Cluster 4	2	1	2	5
	Cluster 5	1	-	2	3
	**Average**	**1.5**	**1**	**1.3**	**3.8**
MexY	Pre-MD	2	2	-	4
	Cluster 1	2	-	2	4
	Cluster 2	3	1	1	5
	Cluster 3	3	1	2	6
	Cluster 4	2	-	2	4
	Cluster 5	2	-	-	2
	**Average**	**2.3**	**0.7**	**1.2**	**4.2**

2.3 Supplementary References

Ramaswamy, V.K., Vargiu, A.V., Malloci, G., Dreier, J., and Ruggerone, P. (2017). Molecular Rationale behind the Differential Substrate Specificity of Bacterial RND Multi-Drug Transporters. *Sci. Rep.* 7(1)**,** 8075. doi: 10.1038/s41598-017-08747-8.

Rice, P., Longden, I., and Bleasby, A. (2000). EMBOSS: the European molecular biology open software suite. *Trends Genet.* 16(6)**,** 276-277. doi: 10.1016/S0168-9525(00)02024-2.

Schrödinger (2015). The PyMOL Molecular Graphics System, Version 1.5 LLC. New York, NY: Schrödinger

Sennhauser, G., Bukowska, M.A., Briand, C., and Grütter, M.G. (2009). Crystal structure of the multidrug exporter MexB from Pseudomonas aeruginosa. *J. Mol. Biol.* 389(1)**,** 134-145. doi: 10.1016/j.jmb.2009.04.001.
